# Diversity and distribution of amino acid decarboxylase enzymes in the human gut bacteria—a bioinformatics investigation

**DOI:** 10.3389/fmicb.2025.1616635

**Published:** 2025-09-17

**Authors:** Matthew Sandoval, Dhara D. Shah

**Affiliations:** ^1^Biodesign Center for Fundamental and Applied Microbiomics, Arizona State University, Tempe, AZ, United States; ^2^School of Mathematical and Natural Sciences, Arizona State University, Glendale, AZ, United States

**Keywords:** human gut bacteria, biogenic amines (BA), amino acid decarboxylase, microbiome, glutamate decarboxylase (GAD), arginine decarboxyalse, lysine decarboxylase, histidine decarboxylase

## Abstract

Biogenic amines play numerous biological functions that include neuromodulation, maintenance of the gut health and motility, gastric acid secretion, regulation of immune response, cell growth, and gene expression. Therefore, it is crucial to comprehend the potential modulation of these molecules by the human gut microbiota. A primary pathway for the generation of these molecules involves the decarboxylation of amino acids, a process facilitated by enzymes known as amino acid decarboxylases (AADCs). Here, we conducted a bioinformatic analysis to understand diversity and prevalence of AADCs from the most prevalent members of the human gut microbiome. This study aims to understand how human gut microbes generate metabolites that influence health and disease, through specific enzyme activities. Our results indicate that AADCs are most abundant in the prominent gut microbial genera, namely *Bacteroides*, *Parabacteroides*, *Alistipes*, and *Enterococcus*. Within these, *Enterococcus faecalis* harbors the most variety of amino acid decarboxylases, potentially playing an important role in driving decarboxylation chemistry in the human gut. Furthermore, among AADCs, arginine decarboxylases are the most common, present in approximately 60% of the frequently found members of the human gut microbiome, followed by aspartate 1-decarboxylases and glutamate decarboxylases. In addition, our sequence analyses of various AADCs demonstrated that a tetrad of amino acids in the PLP binding motif can provide functional identification for AADCs. We hypothesize that the diversity in AADCs and the microbes that harbor them has the potential to alter host metabolic outputs. This could provide a mechanism to use specific changes in microbial genera or species to understand possible metabolite modulations that might influence biological functions. Such studies could lay the groundwork for developing future disease markers or therapeutic interventions.

## Introduction

**Figure 1:**
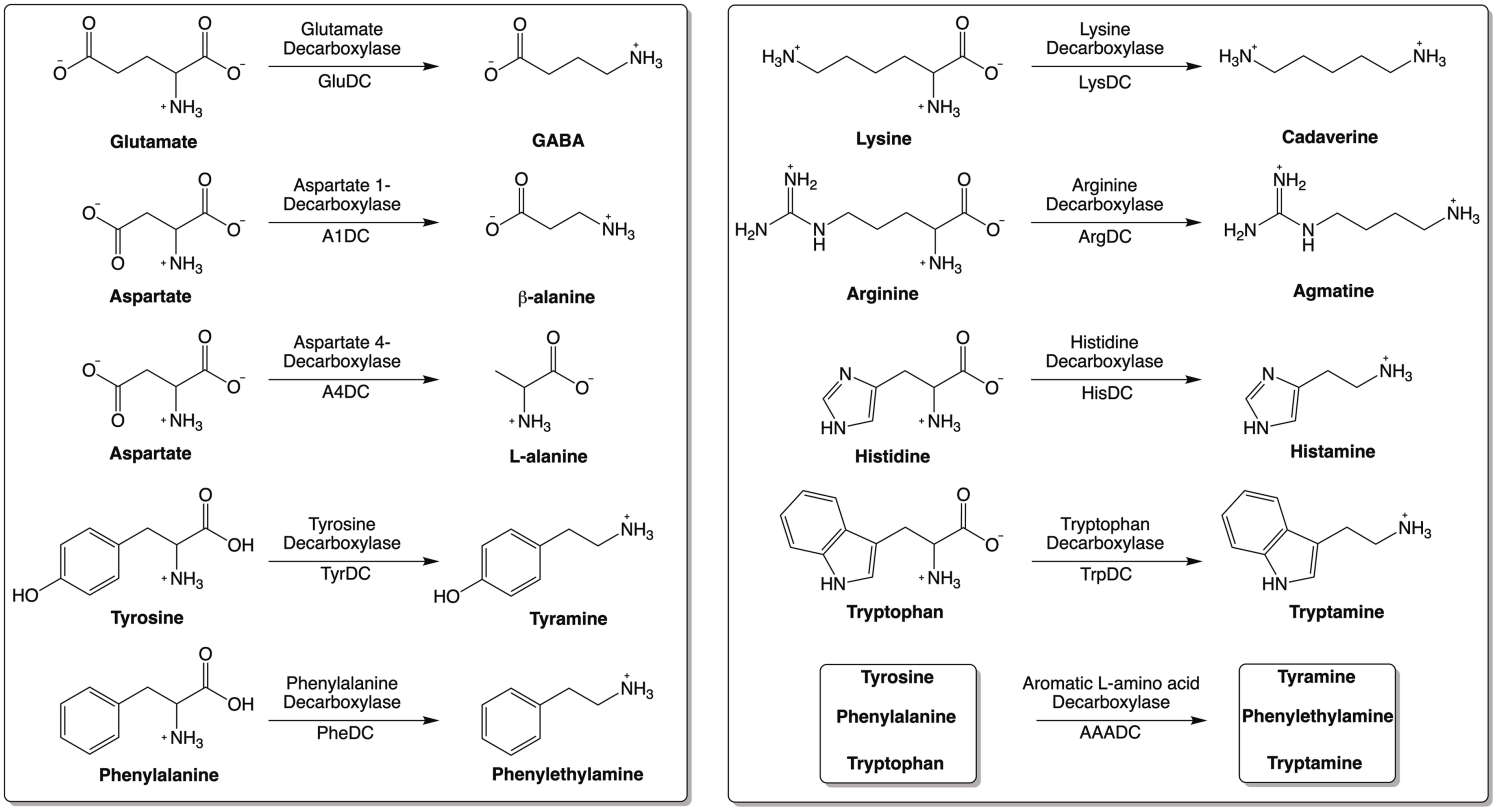
Reactions catalyzed by various amino acid decarboxylases (AADCs).

Amino acid decarboxylases (AADCs) catalyze the decarboxylation of amino acids to generate corresponding amines (Figure 1). Amines produced by these reactions are structurally and functionally diverse. Specifically, the production of neuromodulatory molecules like histamine, tyramine, tryptamine, dopamine, serotonin, and γ-aminobutyric acid (GABA) is dependent on the actions of AADCs (Sugiyama et al., 2022; Duranti et al., 2020; Strandwitz et al., 2019; Wang and Lee, 2007; Wuthrich et al., 2017; Akhova et al., 2021; Burrell et al., 2010; Williams et al., 2014; Maini Rekdal et al., 2019; van Kessel et al., 2019). Apart from their role in the production of multiple neuromodulatory molecules, AADCs are also involved in the biosynthesis of polyamines like spermine, spermidine, putrescine, and cadaverine (Gevrekci, 2017; Tabor and Tabor, 1985). Several studies have investigated the modulations of metabolites produced by amino acid decarboxylases (AADCs), particularly those derived from human gut microbial activity. For example, polyamines are produced by the gut microbiota in the large intestine (Matsumoto et al., 2012; Milovic, 2001) and microbes utilize these for cell growth, during the stress response, and for survival (Gevrekci, 2017). Microbially produced polyamines are also beneficial to the host’s gut health by promoting epithelial renewal, longevity, and recovery of injured mucosa (Nakamura et al., 2021; Tofalo et al., 2019). It is also known that in certain gut microbes, the acidification of the gut environment induces polyamine biosynthesis as a coping method for the acidic stress (Kitada et al., 2018). In an earlier study, tryptamine production was demonstrated to be dependent on tryptophan decarboxylases of members of the human gut microbiota (Williams et al., 2014). Additional studies showed production of other neuromodulatory molecules like serotonin, tyramine, and GABA generated by the actions of various AADCs present in the human gut bacteria (Sugiyama et al., 2022; Maini Rekdal et al., 2019; van Kessel et al., 2019; Otaru et al., 2021). GABA and agmatine which are decarboxylated products of L-glutamate and L-arginine, also combat acidic stress in bacteria (De Biase and Pennacchietti, 2012; Iyer et al., 2003). In fact, AADCs like glutamate and arginine decarboxylases are known to play a role in acid resistance mechanisms present in many prokaryotic organisms by consuming protons during decarboxylation, increasing the pH, and preventing acidic damage to the organism (De Biase and Pennacchietti, 2012; Iyer et al., 2003). Additionally, some polyamines induce glutamate decarboxylase dependent acid resistance systems (Chattopadhyay and Tabor, 2013). These are some of the crucial functions played by the products of AADCs both in the microbes and in the host.

Moreover, during catalysis by AADCs, carboxylic acid groups are removed from amino acids and released as either CO_2_ gas or as dissolved CO_2_. Depending on the form of the CO_2_ released during the reaction, a variety of physiological changes can occur. As a part of a bicarbonate buffering system, it can help maintain the pH within the gut or in the blood stream. In contrast, excessive production of CO_2_ gas can cause discomfort and bloating in humans. Given the physiological significance of the products generated by amino acid decarboxylases, characterizing the nature and abundance of these enzymes in the human gut microbiome can offer valuable insights into microbial metabolism, intermicrobial communication, and host–microbe interactions. While AADC derived metabolites are known to influence host and microbial physiology, this study focuses specifically on the biochemical and sequence-based diversity of AADCs in gut bacteria. The aim is to provide a foundational framework for future investigations rather than to draw direct ecological or health-related conclusions.

## Methods

### Database

This research was conducted using the Integrated Microbial Genomes & Microbiomes (IMG/M) public facing database for genome datasets (Chen et al., 2023).

### Selection of gut microbial candidates

Human gut bacterial candidates were selected from a previously published human gut bacterial genome and culture collection studies by Forster et al. (2019). This study identified human gut microbiota through fecal samples collected from 20 healthy adults from North America and the United Kingdom who had not recently taken antibiotics. Species selection for investigating amino acid decarboxylases was conducted using this data and included human gut bacteria that were present at levels greater than 0.01% within any two samples analyzed as presented in the Supplementary Table S5 from the study conducted by Forster et al. (2019). Species not identified as the member of common human gut microbiota by Forster et al. (2019) were not selected. After species selection, each species was inquired by Taxon ID through IMG/M and alternative species names (recently changed names) were identified and recorded (Supplementary Table S7). The study by Forster et al. was selected due to its systematic characterization of cultured bacterial isolates from the human gut which can enable downstream experimental investigations.

### Enzyme ID (EC number) selection

The IGM/M database was inquired for “decarboxylase” and all amino acid decarboxylases of interest were identified and enzyme IDs were saved (Supplementary Table S8). No exclusion criteria were applied at this stage.

### Extraction of gene ID data harboring amino acid decarboxylases

Each EC number (Enzyme ID) was queried in IMG/M and all gene IDs harboring a specific EC number were exported. All bacteria with an annotated amino acid decarboxylase of interest were included and no exclusion criteria were applied. All bacteria (present in the IMG/M database) containing various amino acid decarboxylases were grouped together under a specific Enzyme ID (Supplementary File S1).

### Refinement of gene ID data with the available abundant human gut microbes

The gene ID data for each EC number (Enzyme ID) was filtered for only those that corresponded to a common human gut bacterial species (or alternative species name as outlined in species selection) selected from the Forster et al. (2019) data as mentioned above. Given the quantity of gene IDs from each species due to the presence of multiple strains, a representative gene ID was selected from the collection with preference for complete genomes present in either ATCC, NCTC, or DSM culture collections. When multiple annotations for a species’ decarboxylase were present in ATCC, NCTC, or DSM, the representative was selected without preference. For species without an annotated genome in ATCC, NCTC, or DSM, an alternative gene ID was selected as the representative without preference (Supplementary File S2).

### Representative species selection

Within each decarboxylase group, one species was selected to have all strains (with different Gene IDs) harboring the decarboxylase enzyme evaluated highlighted in green under each Enzyme ID in the Supplementary File S2. This was necessary to confirm that the selection process for a representative strain for each species without preference, outside of ATCC, NCTC, or DSM annotations, was adequate to become representative for strains within one species. Amino acid sequence homology within the decarboxylase enzyme from various strains of the same species were very high, well above 90%, in almost all cases (Supplementary Table S9). This homology between strains of the same species indicates that the selection of one strain per species was an appropriate method for identifying decarboxylase sequences to be analyzed. There was only one case where the sequence homology was poor. It was found within the tryptophan decarboxylases of *N. alkaliphilus* strains which might be indicative of greater variation within *N. alkaliphilus* enzymes. Specially because annotations for these enzymes are either aromatic amino acid decarboxylase or glutamate or tyrosine decarboxylase. In such cases, without the biochemical characterization of these enzymes, discerning functions will not be possible.

### Multiple sequence alignment

After the identification of a gene ID for each species, the amino acid sequence data for the protein product of each gene ID was exported from IMG/M and saved for further analysis in Supplementary File S3. All amino acid decarboxylase sequences were obtained from IMG/M. The sequence data for each decarboxylase was saved as a FASTA file with the gene ID and species name in the header. Multiple Sequence Alignment was then performed on each group of decarboxylases using ClustalW Omega 1.2.4 with default parameters to evaluate homology of the various human gut microbial amino acid decarboxylases. Alignment outputs were saved, and the Percent Identity Matrix (PIM) file was exported and formatted in Microsoft Excel to visualize similarity between decarboxylases across genera and species.

### Identification of PLP (pyridoxal phosphate) binding motifs within each amino acid decarboxylase group

The PLP Binding motif was identified through the common motif found in many PLP dependent decarboxylases (Momany et al., 1995) from the multiple sequence alignment data. The motif was uniquely identified by its position within the peptide sequence and a characteristic Lysine (K) residue (Supplementary Table S6).

## Results and discussion

### Arginine decarboxylases are the most abundant amino acid decarboxylases (AADCs) in the prevalent members of the human gut microbiome

**Figure 2:**
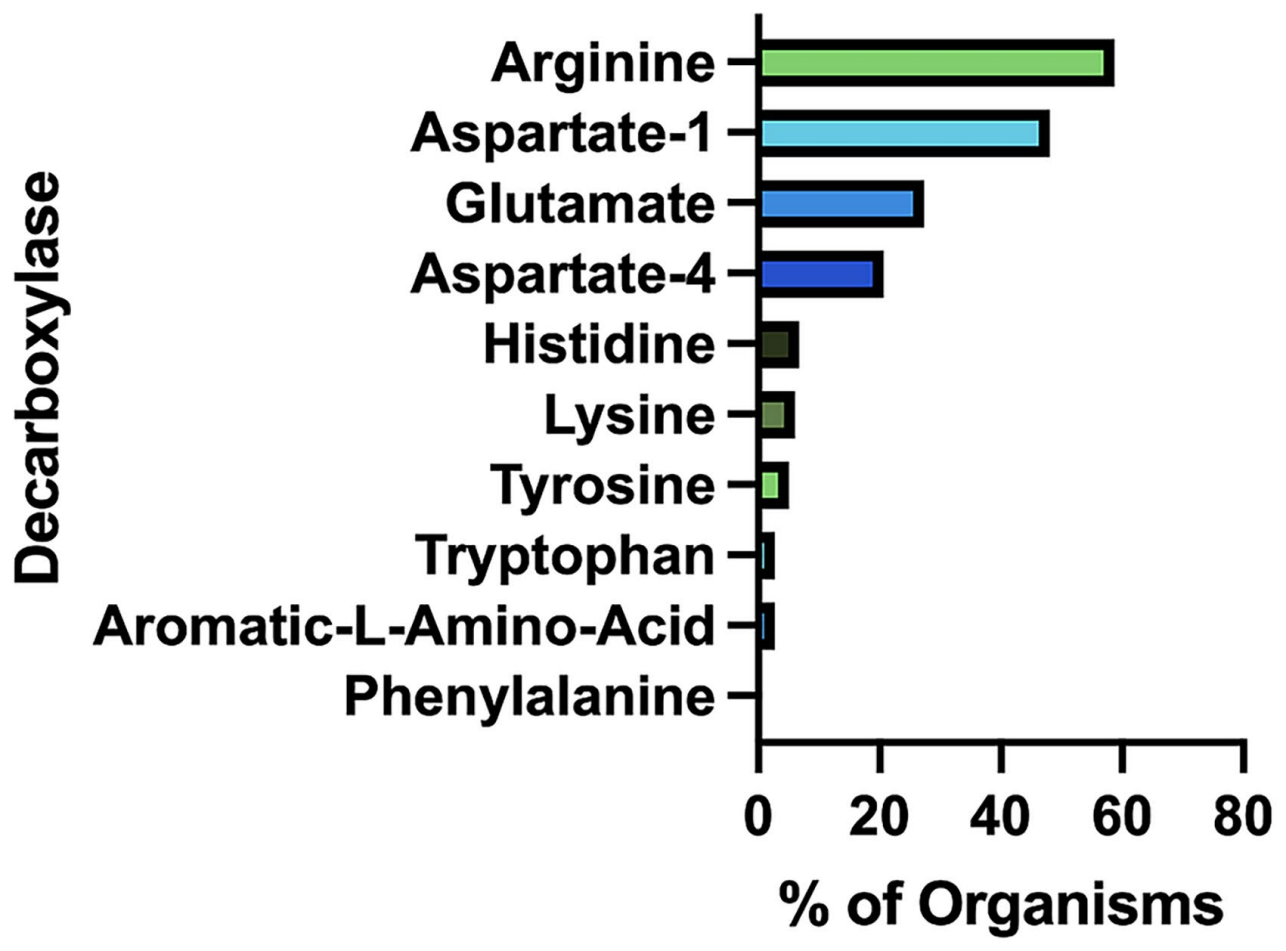
Percent of prevalent human gut bacteria with each type of amino acid decarboxylase (AADC).

Our bioinformatics analysis revealed that arginine decarboxylases were the most represented amino acid decarboxylases among human gut microbiota (Figure 2 and Supplementary Table S1). Around 60% of the commonly found gut bacteria harbor arginine decarboxylases (ArgDCs) followed by aspartate-1 decarboxylases (A1DCs) that were present in approximately 50% of the prevalent human gut bacteria (Figure 2). Based on the annotations, these AADCs are predicted to decarboxylate L-arginine and L-aspartate to produce agmatine and β-alanine (Figure 1), respectively. Agmatine is regulator of polyamine biosynthesis and is a precursor for polyamines like putrescine, spermidine and spermine (Tabor and Tabor, 1985). Gut microbes have a metabolic pathway that includes a conserved arginine decarboxylase and a set of other enzymes for the formation of the most abundant polyamines in the gut, spermidine from agmatine (Sugiyama et al., 2017). In addition to polyamine biosynthesis, ArgDCs play a crucial role in acid resistance and thus help microbes in the survival under extreme acidic conditions. They facilitate protection by utilizing protons during catalysis and increasing the pH of the solution (Burrell et al., 2010). A1DCs, on the other hand, are important in the formation of β-alanine which is a precursor for the biosynthesis of pantothenate that is utilized in the formation of coenzyme A (CoA), an important intermediate in various metabolic pathways (Lopez-Samano et al., 2020). *β*-alanine has also been shown to provide protective effects in individuals with cognitive deficits (Hata et al., 2019; Ostfeld et al., 2023).

The next prominent class of AADCs were glutamate decarboxylases (GluDCs), found to be present in around 27% of the commonly found gut bacteria (Figure 2). Glutamate decarboxylases are known to play an important role in acid resistance mechanism similar to arginine decarboxylases (Damiano et al., 2015). GluDCs catalyze the conversion of L-glutamate to γ-aminobutyric acid (GABA) (Figure 1). GABA is the major inhibitory neurotransmitter in the human central nervous system (McCormick, 1989) whereas microbes utilize GABA as an energy source and certain gut microbes grow solely in the presence of GABA (Strandwitz et al., 2019). With these various functions, glutamate decarboxylases are important AADCs present in the gut microbes. We hypothesize that such overlapping acid resistance mechanisms provided by AADCs like ArgDCs and GluDCs possibly work together in gut microbes. The ability to decarboxylate different amino acid substrates might allow these microbes to survive in various acidic and substrate limiting environments. This strategy allows for continuous acid resistance in microbes specially when one of the pathways become less functional. Following GluDCs in abundance are aspartate-4 decarboxylases (A4DCs). 21% of the gut bacteria contained asparatate-4 decarboxylases (Figure 2). A4DCs catalyze the conversion of L-aspartate to L-alanine (Figure 1) and hence A4DCs are important in the metabolism of these two amino acids (Wang and Lee, 2007). Moreover, we found that histidine (HisDCs) and lysine decarboxylases (LysDCs) were present in around 7 and 6% of the prevalent human gut bacteria, respectively, (Figure 2). Histidine decarboxylases (HisDCs) catalyze the conversion of L-histidine to histamine whereas lysine decarboxylases (LysDCs) catalyze the conversion of L-lysine to cadaverine (Figure 1). Histamine plays an important role in communication of immune responses and as a neuroimmune modulator in the gut (De Palma et al., 2022). In contrast, cadaverine produced by the gut microbes can have both harmful and beneficial effects on the host (Del Rio et al., 2019; Kovacs et al., 2019). In some microbes, cadaverine appears to reduce susceptibility to certain antibiotics (Akhova et al., 2021) while in others, specifically those that produce siderophores, it serves as a precursor in siderophore biosynthesis (Burrell et al., 2012).

Next, to HisDCs and LysDCs, 5% of the prevalent human gut bacteria contained tyrosine decarboxylases (Figure 2). Tyrosine decarboxylases (TyrDCs) catalyze the conversion of L-tyrosine to tyramine (Figure 1). In addition, TyrDCs can also covert L-DOPA to dopamine (Maini Rekdal et al., 2019; van Kessel et al., 2019). Tyramine is a trace amine and is known to displace catecholamine neurotransmitters like dopamine, epinephrine and norepinephrine from pre-synaptic vesicles and interferes with their signaling (Raiteri et al., 1977). Dopamine is a neurotransmitter which is important for motivation, reward, cognition, and motor control (Klein et al., 2019). Lastly, tryptophan decarboxylases (TrpDCs) and aromatic L-amino acid decarboxylases (AAADCs) were equally represented and found in around 3% of the commonly found human gut bacteria (Figure 2). TrpDCs catalyze the conversion of L-tryptophan to tryptamine whereas AAADCs catalyze conversion of aromatic amino acids (tyrosine, tryptophan and phenylalanine) to their corresponding aromatic amines (Figure 1). Tryptamine acts as a neuromodulator in mammalian brain and serves as a regulator of gastrointestinal motility (Williams et al., 2014). A derivative of tryptamine, 5-hydroxytryptamine commonly known as serotonin is also an important neurotransmitter (Berger et al., 2009). AAADCs show broad substrate specificity and other than catalyzing the decarboxylations of three proteinogenic aromatic amino acids, these are known to also decarboxylate derivatives of aromatic amino acids like 3,4-dihydroxyphenylalanine (L-DOPA) (Maini Rekdal et al., 2019; van Kessel et al., 2019) and 5-hydroxytryptophan to produce dopamine and 5-hydroxytryptamine (serotonin)(Luqman et al., 2018). We did not find separately annotated phenylalanine decarboxylases (PheDCs) in any of the common gut microbes. We hypothesize that if there are enzymes which decarboxylate phenylalanine, then these are found under the bigger class of enzymes called aromatic L-amino acid decarboxylases (Sugiyama et al., 2022). The decarboxylation product of L-phenylalanine is phenylethylamine (PEA) (Figure 1). PEA like many other trace amines can bind to trace amine-associated receptor 1 and impart various physiological effects (Babusyte et al., 2013; Xie and Miller, 2008). Some of these are, activation of blood leukocytes and alteration of monoamine transporter function in the brain (Xie and Miller, 2008). Given the diverse biological roles of the products generated by AADC-mediated reactions, gaining insight into their distribution among human gut microbes may inform future methods for modulating concentrations of these compounds in humans.

### The prevalent gut microbial genus *Bacteroides* has the highest abundance of amino acid decarboxylases (AADCs)

**Figure 3:**
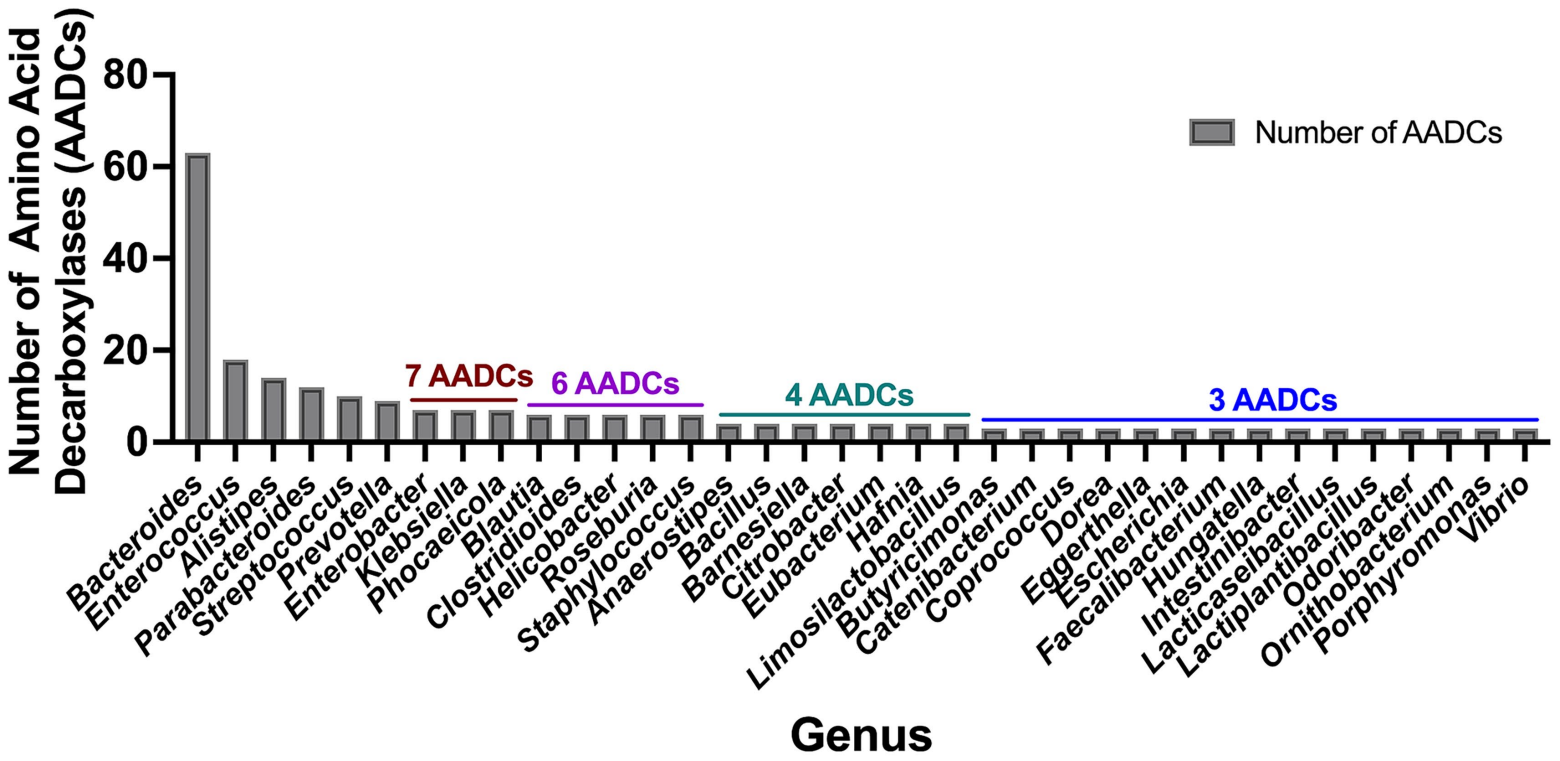
Total number of amino acid decarboxylases (AADCs) at genus level. The plot depicts genus level occurrences of different classes of amino acid decarboxylases in the prevalent human gut bacteria. The breakdown of number of species within each genus is presented in Supplementary Table S1. For the genera included within this figure the total number of species within each genus is: *Bacteroides* total 18, *Enterococcus* total 5, *Alistipes* total 5, *Parabacteroides* total 3, *Streptococcus* total 20, *Prevotella* total 6, *Enterobacter* total 2, *Klebsiella* total 2, *Phocaeicola* total 2, *Blautia* total 4, *Clostridioides* total 1, *Helicobacter* total 3, *Roseburia* total 3, *Staphylococcus* total 4, *Anaerostipes* total 2, *Bacillus* total 1, *Barnesiella* total 1, *Citrobacter* total 1, *Eubacterium* total 2, *Hafnia* total 1, *Limosilactobacillus* total 1, *Butyricimonas* total 1, *Catenibacterium* total 1, *Coprococcus* total 2, *Dorea* total 2, *Eggerthella* total 1, *Escherichia* total 1, *Faecalibacterium* total 1, *Hungatella* total 1, *Intestinibacter* total 1, *Lacticaseibacillus* total 2, *Lactiplantibacillus* total 2, *Odoribacter* total 1, *Ornithobacterium* total 1, *Porphyromonas* total 1, and *Vibrio* total 1. Only genera harboring ≥ 3 AADCs are included here. The entire list for genus level AADCs occurrences are presented in Supplementary Table S2.

In our investigation of annotated amino acid decarboxylases among common human gut bacteria, we observed a broad spectrum of abundance levels for various classes of amino acid decarboxylases (AADCs) within each genus, as shown in Figure 3. The detected numbers varied widely, from as many as 60 to as few as none. Our analysis showed that the genus *Bacteroides* contained the highest number of AADCs (Figure 3). We found 63 annotated AADCs in the members of *Bacteroides* genus which were significantly higher than any other gut microbial genus (Figure 3 and Supplementary Table S2). The next 5 genera after *Bacteroides* showed anywhere between 9 and 18 AADCs. These were *Enterococcus* with 18, *Alistipes* with 14, *Parabacteroides* with 12, *Streptococcus* with 10, and *Prevotella* with 9 AADCs (Figure 3 and Supplementary Table S2). All the top 6 genera belong to the two phyla Bacteroidetes and Firmicutes, which are also the dominant CO_2_ producing phyla and together constitute over 90% of the total microbial population in the human gut (Mutuyemungu et al., 2023). The next three genera *Enterobacter* (Proteobacteria), *Klebsiella* (Proteobacteria), and *Phocaeicola* (Bacteroidetes) each contain total 7 AADCs (Figure 3 and Supplementary Table S2). There were 5 genera that showed the presence of 6 AADCs, 7 genera that had 4 AADCs, and 15 genera that had 3 AADCs as highlighted in Figure 3. Apart from that, 48 genera contained either 1 or 2 AADCs and 5 genera did not have any AADCs (Supplementary Table S2). *Bacteroides* represents one of the most prevalent genera within the human gut microbiome, leading to a higher number of its species and strains being well characterized relative to other genera. Within the gut microbes examined in this study, multiple *Bacteroides* species are found in significant numbers. Therefore, the results presented here may be influenced by the extensive species data from *Bacteroides*, as compared to data from other microbial genera in the human gut.

### *Enterococcus faecalis* harbors the most variety of amino acid decarboxylases

**Figure 4:**
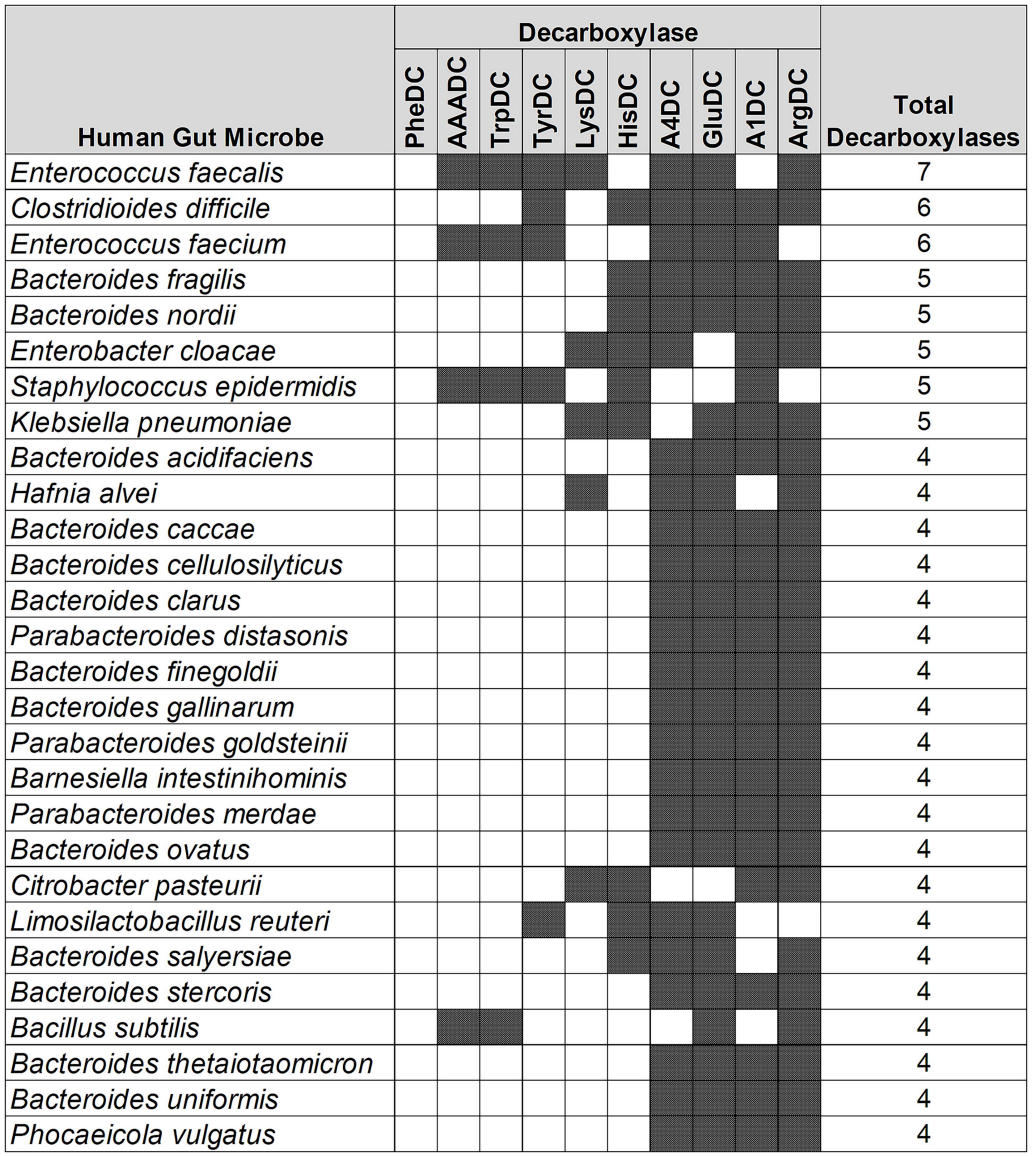
Different classes of AADCs present in the most commonly found human gut bacteria. The figure represents prevalent human gut bacteria harboring different classes of AADCs. Here, shaded areas correspond to the presence of AADCs, and non-shaded (blank white) areas correspond to the absence of AADCs. The figure only includes bacteria containing at least 4 different classes of AADCs. The full list of the bacteria containing various classes of AADCs can be found within Supplementary Table S3.

Next, we performed an analysis to understand if there are any patterns in the type of AADCs present in the prominent gut microbes. Our results demonstrated that the type of AADCs present were not genus specific and different species within the same genus can contain different classes of AADCs (Figure 4 and Supplementary Table S3). We found that *Enterococcus faecalis* had the most variety of AADCs. It contained 7 out of 10 AADCs analyzed in this study. However, the genus *Enterococcus* represented anywhere from 1 to 7 types of AADCs at the species level (Figure 4 and Supplementary Table S3).

**Figure 5:**
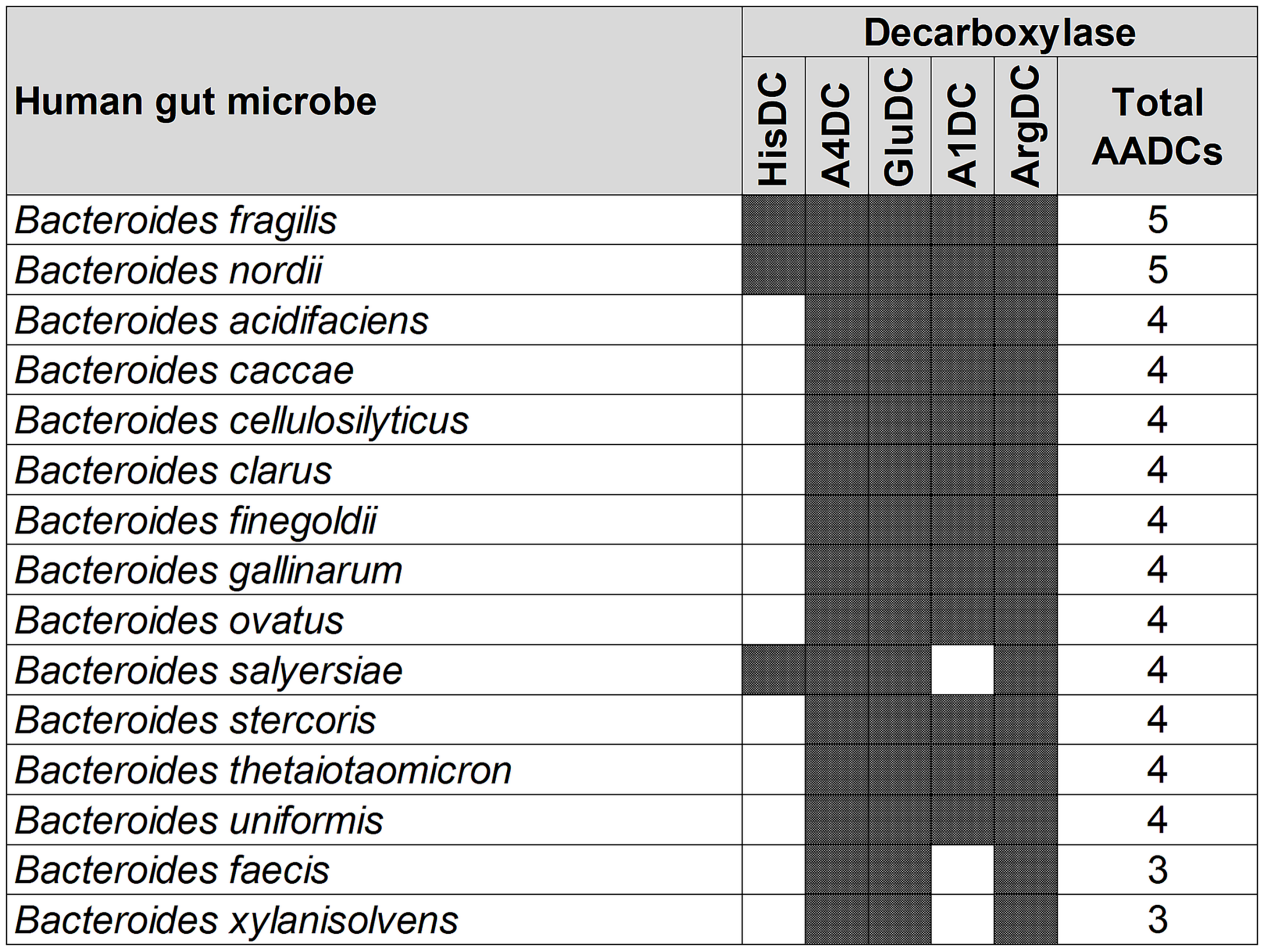
Different classes of AADCs present in various species of the genus *Bacteroides.* The figure depicts the presence of different classes of AADCs in various species of the genus *Bacteroides*. The shaded areas in the figure show the presence of AADCs, and non-shaded (blank white) areas show the absence of AADCs.

A similar variation, but not as large, was also seen with the genus *Bacteroides*. We found that the various species of *Bacteroides* contained 3 to 5 different classes of AADCs where the majority of the species had 4 different types of AADCs (Figure 5). It is interesting to note that the genus *Bacteroides* showed the presence of at least 3 classes of AADCs which points towards the importance of AADCs in their metabolism. Figure 5 illustrates the distribution of different AADCs across various *Bacteroides* species. All species analyzed in this study have GluDC, ArgDC, and A4DC. In contrast, A1DCs and HisDCs were detected in only a subset of species. Specifically, HisDCs are the least abundant, present in only three *Bacteroides* species. Additionally, we observed that the genus *Bacteroides* lacks AADCs that are able to catalyze decarboxylations of aromatic amino acids – tyrosine, tryptophan, and phenylalanine. These reactions are generally catalyzed by TyrDC, TrpDC, PheDC, and AAADC. This points towards the inability of the genus *Bacteroides* to produce aromatic amines. Moreover, while most studies to date including ours suggest that *Bacteroides* species generally lack the ability to produce aromatic amines via the canonical decarboxylation of aromatic amino acids (Sugiyama et al., 2022), there are two notable exceptions. Horvath et al. (2022) and Fernandez-Cantos et al. (2024) reported tyramine production in various *Bacteroides* species, including *B. ovatus*. However, neither study identified annotated tyrosine decarboxylases in these organisms which is consistent with our own findings. This suggests that if *Bacteroides* are capable of producing tyramine, they likely do so via alternative non-canonical pathways. Supporting this, Horvath et al. attributed tyramine production in *B. ovatus* to the activity of an annotated aspartate-1-decarboxylase rather than a dedicated tyrosine decarboxylase. In addition, while tyramine was detectable in *B. ovatus* cultures *in vitro*, the same study did not observe any significant changes in tyramine levels *in vivo* in colonized mice, suggesting limited physiological relevance under those conditions. Together, these findings support our conclusion that *Bacteroides* generally do not utilize the classical aromatic amino acid decarboxylation pathway to produce aromatic amines and may instead rely on alternative or less efficient mechanisms which remain to be fully elucidated. In addition to the absence of AAADCs, *Bacteroides* also lack annotated lysine decarboxylases (LysDCs), suggesting a limited capacity for cadaverine biosynthesis via the canonical decarboxylation pathway.

Supplementary Table S3 represents a full list of prevalent human gut microbes with or without the presence of 10 classes of AADCs. In addition to microbes harboring 4 or more AADCs depicted in Figure 4, there were 50 bacteria containing at least one type of AADC, 40 bacteria with two different types of AADCs, and 20 bacteria with 3 different types of AADCs (Supplementary Table S3). Moreover, we found that the genus *Bifidobacterium* severely lacked AADCs. Other than the one species—*B. adolescentis* with one AADC which is GluDC (Duranti et al., 2020), none other members of the genus had any of the 10 AADCs. During our analysis, we observed variability at the strain level in a few instances among identical species of the microbes. The scope of this study is beyond the strain level variation. However, our results indicated that the types of AADCs can vary at the genus, species, and/or strain level. This variability in AADCs can potentially affect microbial metabolism, specifically the ability to generate biogenic amines including trace amines, polyamines, and neuromodulatory and immunomodulatory molecules like GABA and histamine with varying biological functions either towards the community of gut microbes or towards the host. In addition, this variability might drive variations in the production of carbon dioxide that can impart either advantageous or detrimental effects to the host some of which are impacting pH balance, changes in gut motility, and bloating due to gas accumulation.

### Arginine decarboxylases (ArgDCs) display the most significant differences in length among the human gut bacteria

To understand if the AADCs found in various gut microbes are similar or different, we conducted a protein length analysis on all the AADCs found in the common human gut bacteria (Supplementary Table S4). Through this analysis, we found that TyrDCs and A1DCs shared similar length across different genera and species (Table 1). These were followed by HisDCs and GluDCs. AADCs from these two classes show variability of around 80 to 90 residues in the protein length across prevalent human gut microbial genera and species. However, A4DCs, AAADCs, and TrpDCs showed larger variation in the length which was from 120 to 180 residues. Our dataset with AAADCs and TrpDCs is very small that contains total five enzymes. Interestingly, AAADCs and TrpDCs were exactly the same which points towards two possible annotations provided as AAADCs and TrpDCs. LysDCs exhibited an even broader range of protein lengths spanning approximately 300 residues with observed lengths ranging from 450 to 740 amino acids (Table 1). A study by Li et al. (2021) on LysDC evolution identified two distinct forms: an ancestral shorter variant typically 480–490 amino acids in length, and an extended form ranging from 710–755 amino acids. This is consistent with our findings which possibly include both forms of LysDCs. The presence of both length classes in our dataset suggests that gut microbial LysDCs may have evolved along divergent structural lineages, potentially reflecting functional divergence or adaptation to distinct ecological niches within the gut environment. The arginine decarboxylases (ArgDCs) displayed the greatest variability in protein length among the enzymes analyzed. The lengths ranged from 154 to 790 amino acids, with most proteins clustering around a mode of around 630 residues (Table 1). This heterogeneity likely reflects the presence of multiple evolutionary lineages of ArgDCs within the human gut microbiome. In prokaryotes, four distinct classes of ArgDCs have been identified (Burrell et al., 2010), and our findings suggest that gut microbes may harbor representatives from more than one class. Specifically, Bacteroidetes encode ArgDCs with an alanine racemase (AR) fold, while Firmicutes predominantly harbor ArgDCs with an aspartate aminotransferase (AAT) fold (Burrell et al., 2010). These folds are characteristic of pyridoxal 5′-phosphate (PLP)-dependent enzymes, with Fold Type I corresponding to the AAT-fold and Fold Type III to the AR-fold (Tran and Brown, 2022). The divergence in structural folds points to independent evolutionary origins of ArgDCs across microbial taxa. Functionally, some ArgDCs are acid-inducible and contribute to acid resistance particularly under gastrointestinal stress conditions, while others are not regulated by pH and are primarily involved in polyamine biosynthesis which supports microbial growth and host–microbe interactions (Burrell et al., 2010). This functional diversity further highlights the adaptive significance of maintaining distinct ArgDC types within the gut microbiota.

**Table 1:** Length variation within amino acid decarboxylases.

AADC	Enzyme ID	Length range	Length max	Length min	Mode	Number of enzymes
ArgDC	4.1.1.19	636	790	154	630	107
LysDC	4.1.1.18	289	739	450	712	12
TrpDC	4.1.1.105	178	513	335	470–484	5
AAADC	4.1.1.28	178	513	335	470–484	5
A4DC	4.1.1.12	121	588	467	547	38
GluDC	4.1.1.15	92	551	459	480	51
HisDC	4.1.1.22	85	380	295	295	11
A1DC	4.1.1.11	29	142	113	117	87
TyrDC	4.1.1.25	20	635	615	625	9

Analysis in this table is based on the amino acid sequence data from Supplementary Table S4.

### Sequence identities within AADCs from each class provide either functional significance or the microbial host specificity

Our analysis presents interesting aspects about decarboxylase enzymes within each group of AADCs. As mentioned above, prokaryotic organisms harbor multiple types of arginine decarboxylases (ArgDCs), each of which is typically specific to a particular phylum (Burrell et al., 2010). This is evident in the percent identity matrix of all ArgDCs, which shows a few regions with high sequence identities (Supplementary Table S5). In the percent identity matrix for the other large group containing GluDCs, we found two major areas with high sequence identities (Supplementary Table S5). The region outlined in yellow predominantly includes genera from the phylum Firmicutes (now Bacillota), which comprises Gram-positive organisms. However, the region outlined in green mostly represents organisms that belong to the phylum Bacteroidetes (now Bacteroidota) with two exceptions of *Faecalibacterium prausnitzii* and *Catenibacterium mitsuokai*. Based on these results, gut microbial GluDCs from the phylum Bacteroidetes are more similar to each other than to GluDCs of the phylum Firmicutes. When we compare GluDCs of Firmicutes to GluDCs of Bacteroidetes, we still see around 40–50% overall sequence identity which is significant (Supplementary Table S5). In contrast to ArgDCs, where Firmicutes and Bacteroidetes contain entirely different classes of enzymes, the differences observed within GluDCs are more subtle and lie within the overall amino acid sequences. Moreover, in the percent identity matrix of GluDCs, we found two enzymes that are significantly different from the whole group with only 15–20% overall sequence identity to the other members of the group. These were from *Pararheinheimera texasensis* and *Vibrio cincinnatiensis* (Supplementary Table S5). Despite their low sequence identity, GluDCs from the organisms mentioned above may still retain their enzymatic functions if key active site residues are conserved or if they have resulted through convergent evolution. However, such low sequence identity also suggests the potential for functional divergence, indicating that these enzymes may carry out distinct biochemical roles and belong to a separate class of AADCs—a hypothesis that requires experimental validation. We saw a similar pattern with A1DCs where we observed three distinct areas with high sequence identities. These areas are highlighted in Supplementary Table S5, where the top left most (highlighted in yellow) contain organisms from the phylum Proteobacteria (now pseudomondota) which are primarily Gram-negative and facultative anaerobes. The middle area with high sequence identity (highlighted in green) was occupied mostly by the members of the phylum Firmicutes (now Bacillota) which are Gram-positive and anaerobic organisms. We did see some exceptions in that area. The last area with the high sequence identity was the right bottom area (highlighted in cyan) that represented members of the phylum Bacteroidetes (now Bacteroidota), which are Gram-negative and anaerobic organisms.

A4DCs are less prevalent than the other AADC classes discussed above. The percent identity matrix for A4DCs, reveals a distinct region of high sequence identity corresponding to members of the phylum Bacteroidetes (highlighted in cyan) (Supplementary Table S5). Additionally, two groups of AADCs contain the exact same members and proteins. These two are AAADCs and TrpDCs (Supplementary Table S5). Interestingly, these enzymes were mostly found within the members of the phylum Firmicutes (now Bacillota). We identified one member of the phylum Actinobacteria (now Actinomycetota), *Nitriliruptor alkaliphilus* harboring AAADC/TrpDC. Of the other smaller groups of AADCs, HisDCs were present equally in both phyla, Bacteroidetes and Firmicutes (Supplementary Table S5). The percent identity matrix (Supplementary Table S5) displayed two HisDCs sharing only limited sequence identity with the remaining of the group. These were present in Proteobacteria, *Enterobacter cloacae* and *Klebsiella pneumoniae*. The overall sequence identity of these two with the whole group of HisDCs is only around 14–20%. This presents a similar case as seen previously with glutamate decarboxylases displaying low sequence identities compared to the rest of GluDCs. The next AADCs class that is similar in prevalence as HisDCs is LysDCs. We found that LysDCs are mostly present in the members of the phylum Proteobacteria (now Pseudomonadota) with only two members from the phylum Firmicutes (now Bacillota) (Supplementary Table S5). In addition, LysDCs showed very high sequence identity within the group with the average percent identity of around 70% (Table 2). However, a LysDC from *Peptoniphilus harei* was found to be very different compared to the remaining of the group with an overall sequence identity of only 25–27% (Supplementary Table S5). This analysis suggests that the enzyme may not carry out the predicted function and could instead belong to a different subclass of AADCs or it has diverged evolutionarily from all other LysDCs. However, an experimental validation will be necessary to identify its true function. The last group of AADCs that is smaller in size is TyrDCs. Our analysis revealed that TyrDCs are mostly present in the members of the phylum Firmicutes (now Bacillota) (Supplementary Table S5). We found only one enzyme that was from an Actinobacteria, *Cutibacterium acnes*. TyrDC from this organism showed the lowest sequence identity of around 42–45% to all the other members of the group. This class exhibited the greatest sequence identity as a group when contrasted with any other group of AADCs (Table 2).

**Table 2:** Percent identity based on the amino acid sequence similarities within each group of AADCs.

Amino acid decarboxylase	Average percent identity	Minimum percent identity
Arginine Decarboxylase	N/A	N/A
Tryptophan Decarboxylase	50.86	24.84
Histidine Decarboxylase	44.08	30.34
Aromatic-L amino acid Decarboxylase	50.86	24.84
Glutamate Decarboxylase	55.73	36.03
Aspartate-1 Decarboxylase	58.68	31.97
Aspartate-4 Decarboxylase	57.14	35.58
Lysine Decarboxylase	69.44	61.88
Tyrosine Decarboxylase	72.18	42.83

### A tetrad of amino acids in the PLP binding motif can provide functional identification and assignment for most amino acid decarboxylases (AADCs)

Next, we explored the extent of variation among these AADCs in the regions surrounding their cofactor binding sites. Most known amino acid decarboxylases studied here utilize PLP as the cofactor to catalyze decarboxylation reactions. PLP cofactor binds to amino acid decarboxylases via a strictly conserved lysine residue and this lysine is present in all PLP dependent decarboxylases (Momany et al., 1995). In the absence of a substrate, PLP cofactor forms a schiff base with an amino group of a specific lysine residue in the active site of amino acid decarboxylases. While in the presence of the substrate, the schiff base formation with lysine is then replaced by an amino group of the substrate. A previous study with a lysine decarboxylase from *Selenomonas ruminantium* showed importance of amino acid residues that are present in the close vicinity of the lysine residue involved in the schiff base formation in determining substrate specificity (Takatsuka et al., 2000). For this reason, we decided to understand signature motifs around this important lysine residue.

Our analysis of the partial PLP binding motifs specifically four amino acids surrounding the conserved lysine residue showed that within each group of AADCs, these tetrads are conserved (Table 3). Our examination focusing on the four amino acids around the conserved lysine, revealed that these tetrads are preserved within each AADC group. For GluDCs, we see a conserved amino acid tetrad of “SGHK” (Table 3). Based on our percent identity matrices, we saw that there were two outliers in GluDCs group, *Pararheinheimera texasensis* and *Vibrio cincinnatiensis*. The PLP binding motif analysis for these two is in agreement with the sequence similarity analysis (Supplementary Table S6). We observed that the two outliers had a different PLP conserved tetrad of “DAHK.” These findings support our hypothesis that the annotated GluDCs from *Pararheinheimera texasensis* and *Vibrio cincinnatiensis* may exhibit functional divergence, potentially performing distinct biochemical roles and representing a separate class of AADCs as discussed earlier. Upon examining the PLP binding motifs of other AADCs, we observed that DAHK motif is commonly associated with the AAADCs/TrpDCs family (Table 3 and Supplementary Table S6). The presence of this motif in the GluDCs from *P*. *texasensis and V. cincinnatiensis*, combined with their low sequence identity to other GluDCs, suggests that they may function as aromatic L-amino acid decarboxylases. In addition to our analysis, experimental evidence collected by *in vitro* characterization of purified enzymes can provide correct functions for these AADCs. For the aromatic-L-amino acid decarboxylases group that also contains tryptophan decarboxylases, the conserved tetrad residues with variations found at the first and the second positions were “[DN]-[AP]-HK,” where we find motifs like DAHK, DPHK, NPHK, and NAHK (Table 3 and Supplementary Table S6). These variations might be useful in understanding specificity of AAADCs towards various aromatic amino acids. One such example is in Table 3, where we see a conserved tetrad of “DPHK” for TyrDCs. We did not find any exceptions in this conserved motif in annotated TyrDCs (Supplementary Table S6). It is possible that decarboxylases under the group AAADCs harboring DPHK motifs might be TyrDCs. However, this will need a thorough experimental investigation. For A4DCs, we found conserved tetrad residues as “S-[Fy]-[Sa]-K” with variations found at the first and the second positions (Table 3). The primary and most commonly found motif was “SFSK,” followed by “SYSK” found in only 5.3% of enzymes of this class and lastly the motif “SFAK” found in only 2.6% enzymes of this class (Table 3 and Supplementary Table S6). The next group of enzymes harboring conserved tetrad was LysDCs. We found a conserved tetrad of “N-[Ct]-HK” with variations observed at the second position (Table 3). Here, the most found motif was “NCHK.” In only one instance, we found the alternate motif of “NTHK” (Supplementary Table S6). However, this motif was present in an enzyme from *Peptoniphilus harei* that was an outlier during our sequence identity analysis. The motif is also present at a different position (residues 99–102) compared to all the other enzymes in the group which showed motif within amino acid residues 243–246 (254–257 in one case). These observations point towards the possibility of the enzyme from *P. harei* to be AADC from another group or an unusual LysDC. Experimental evidence is required in understanding the true function and nature of this enzyme. All identifiable PLP binding motifs shared the essential lysine (K) residue in similar positions. This residue was most preceded by a histidine (H) residue but was replaced with mostly serine (S) and rarely with alanine (A) resides within the group asparate-4 decarboxylases (A4DCs). Because gut bacteria harbor diverse types of ArgDCs, a single conserved PLP binding motif could not be identified (Table 3).

**Table 3:** PLP binding motif in AADCs of common human gut microbiota.

Amino Acid decarboxylases	PLP motif	Primary motif	Secondary motifs
Glutamate	SGHK	SGHK	N/A
Aspartate-4	S-[Fy]-[Sa]-K	SFSK	SYSK, SFAK
Lysine	N-[Ct]-HK	NCHK	NTHK
Tyrosine	DPHK	DPHK	N/A
Tryptophan/Aromatic-L-Amino Acid	[DN]-[AP]-HK	DAHK	NAHK, DPHK, NPHK
Arginine	Unable to Determine	N/A	N/A
Asparate-1	Pyruvoyl-dependent	N/A	N/A
Histidine	Pyruvoyl-dependent	N/A	N/A

Apart from the PLP dependent amino acid decarboxylases, another class of AADCs are also present in prokaryotes, known as pyruvoyl-dependent amino acid decarboxylases. There are three known AADC groups that contain pyruvoyl cofactor in place of PLP cofactor. These are histidine decarboxylases (HisDCs) (Huynh and Snell, 1985a,b; Recsei and Snell, 1985; Snell, 1986), aspartate 1-decarboxylases (A1DCs) (Nozaki et al., 2012), and some arginine decarboxylases (ArgDCs) (Tolbert et al., 2003). While analyzing length variations among ArgDCs, we identified a subset of very short sequences (<200 amino acids) annotated as ArgDCs. These lacked conserved PLP binding motifs, suggesting that they may be pyruvoyl-dependent enzymes. This is consistent with previous studies reporting that pyruvoyl-dependent ArgDCs typically have protein lengths under 200 amino acids (Tolbert et al., 2003; Giles and Graham, 2007). Similarly, for HisDCs and A1DCs identified in prevalent members of the human gut microbiota, we also did not detect PLP binding motifs, consistent with their classification as pyruvoyl-dependent decarboxylases (Table 3). Unlike ArgDCs, all HisDCs and A1DCs found within this study were all predicted to be pyruvoyl-dependent decarboxylases. Interestingly, human histidine decarboxylase (HisDC) is a PLP dependent enzyme (Komori et al., 2012) whereas HisDCs of the human gut bacteria are pyruvoyl-dependent (Mou et al., 2021). While both types of enzymes produce histamine, the underlying mechanisms differ between human enzymes and those found in gut microbes, which could result in variations in activity and regulatory control. In contrast to HisDCs, humans do not have an aspartate 1-decarboxylase (A1DC) that produces *β*-alanine unlike the members of the human gut microbiome. Humans produce β-alanine via a separate metabolic pathway and not via the direct decarboxylation of L-aspartate (Brown and Williamson, 1982). β-alanine is the precursor for the dipeptide carnosine which is found in muscles and can help combat muscular fatigue during strenuous exercise (Mahootchi et al., 2020). Additionally, β-alanine has been identified as a neurotransmitter and is an essential component of the coenzyme A (Brown and Williamson, 1982; Tiedje et al., 2010). However, recently a mammalian enzyme called GADL1 (glutamic acid decarboxylase like 1) showed a direct decarboxylation activity with L-aspartate to produce β-alanine (Mahootchi et al., 2020). This enzyme is a PLP dependent enzyme in contrast to pyruvoyl-dependent gut microbial enzymes. While PLP dependent enzymes are known to be older and more versatile, pyruvoyl-dependent enzymes provide a streamlined alternative specifically due the ability to produce their cofactor independently.

This study offers a systematic, genome-wide overview of amino acid decarboxylases (AADCs) across gut bacterial species. While experimental validation is needed to confirm these functional annotations, our predictions are reinforced by prior biochemical characterizations of diverse AADCs that support the reliability and significance of our findings (Williams et al., 2014; Horvath et al., 2022; Fernandez-Cantos et al., 2024; Takatsuka et al., 2000; Dadi et al., 2025).

## Conclusion

Our identification of gut microbial species harboring diverse amino acid decarboxylases (AADCs) provides a foundation for *in vitro* biochemical characterization using purified enzymes, as well as functional investigations of these enzymes in both monoculture and co-culture systems to determine their physiological roles in microbial metabolism and community interactions. In addition, the biochemical diversity of AADCs uncovered in our study raises intriguing questions about substrate specificity, regulatory mechanisms, and potential impacts on host physiology, specifically in the context of neuromodulatory and immunomodulatory metabolites. Future studies can also explore the ecological and evolutionary drivers underlying the distribution of AADCs across gut microbes and assess how dietary inputs or host factors may modulate their expression and activity *in vivo*.

## Data Availability

The original contributions presented in the study are included in the article/Supplementary material, further inquiries can be directed to the corresponding author.

## Supplementary material

SUPPLEMENTARY TABLE S1 Percent of microbes with each type of AADC.

SUPPLEMENTARY TABLE S2 Genus level occurrences of AADCs.

SUPPLEMENTARY TABLE S3 Types of AADCs in gut microbes.

SUPPLEMENTARY TABLE S4 Amino acid sequences.

SUPPLEMENTARY TABLE S5 Percent identity matrixes for all AADCs.

SUPPLEMENTARY TABLE S6 PLP motifs of all AADCs.

SUPPLEMENTARY TABLE S7 Taxon ID and alternative names.

SUPPLEMENTARY TABLE S8 Decarboxylase enzymes.

SUPPLEMENTARY TABLE S9 Percent identity in amino acid sequences of each type of decarboxylase from the different strains of the same species.

SUPPLEMENTARY FILE S1 EC ID search raw data.

SUPPLEMENTARY FILE S2 EC ID search gene ID data.

SUPPLEMENTARY FILE S3 Amino acid sequences for decarboxylases.
